# Access and Use of Interventions to Prevent and Treat Malaria among Pregnant Women in Kenya and Mali: A Qualitative Study

**DOI:** 10.1371/journal.pone.0119848

**Published:** 2015-03-23

**Authors:** Jenny Hill, Kassoum Kayentao, Florence Achieng, Samba Diarra, Stephanie Dellicour, Sory I. Diawara, Mary J. Hamel, Peter Ouma, Meghna Desai, Ogobara K. Doumbo, Feiko O. ter Kuile, Jayne Webster

**Affiliations:** 1 Department of Clinical Sciences, Liverpool School of Tropical Medicine, Liverpool, United Kingdom; 2 Malaria Research and Training Centre, University of Sciences, Techniques and Technologies of Bamako, Mali; 3 Kenya Medical Research Institute/Center for Global Health Research, Kisumu, Kenya; 4 Centers for Disease Control and Prevention, Atlanta, Georgia, United States of America and Kenya; 5 Disease Control Department, London School of Tropical Medicine and Hygiene, London, United Kingdom; Institut de Recherche pour le Développement, FRANCE

## Abstract

**Background:**

Coverage of malaria in pregnancy interventions in sub-Saharan Africa is suboptimal. We undertook a systematic examination of the operational, socio-economic and cultural constraints to pregnant women’s access to intermittent preventive treatment (IPTp), long-lasting insecticide-treated nets (LLINs) and case management in Kenya and Mali to provide empirical evidence for strategies to improve coverage.

**Methods:**

Focus group discussions (FGDs) were held as part of a programme of research to explore the delivery, access and use of interventions to control malaria in pregnancy. FGDs were held with four sub-groups: non-pregnant women of child bearing age (aged 15–49 years), pregnant women or mothers of children aged <1 year, adolescent women, and men. Content analysis was used to develop themes and sub-themes from the data.

**Results:**

Women and men’s perceptions of the benefits of antenatal care were generally positive; motivation among women consisted of maintaining a healthy pregnancy, disease prevention in mother and foetus, checking the position of the baby in preparation for delivery, and ensuring admission to a facility in case of complications. Barriers to accessing care related to the quality of the health provider-client interaction, perceived health provider skills and malpractice, drug availability, and cost of services. Pregnant women perceived themselves and their babies at particular risk from malaria, and valued diagnosis and treatment from a health professional, but cost of treatment at health facilities drove women to use herbal remedies or drugs bought from shops. Women lacked information on the safety, efficacy and side effects of antimalarial use in pregnancy.

**Conclusion:**

Women in these settings appreciated the benefits of antenatal care and yet health services in both countries are losing women to follow-up due to factors that can be improved with greater political will. Antenatal services need to be patient-centred, free-of-charge or highly affordable and accountable to the women they serve.

## Introduction

Pregnant women living in malaria endemic areas of sub-Saharan Africa are at substantial risk of the adverse consequences of malaria in pregnancy [1], and each year an estimated 55 million pregnancies occur in areas with stable *P*. *falciparum* malaria [2]. These adverse consequences can be prevented through the use of two highly effective prevention interventions, intermittent preventive treatment with sulphadoxine-pyrimethamine (IPTp-SP) [3] and long-lasting insecticide-treated nets (LLINs) [4]. In areas of stable malaria transmission in Africa WHO recommends a package of intermittent preventive treatment (IPTp) with sulphadoxine–pyrimethamine (SP) and use of insecticide-treated nets (ITNs), together with effective case management of clinical malaria and anaemia [5,6]. Until 2006, WHO recommended two doses of SP for IPTp, taken one month apart commencing after quickening (approximately 18 weeks gestation) [7,8], and together with ITNs, is routinely delivered through antenatal clinics. WHO Antenatal care guidelines recommend four ANC visits during every pregnancy, starting as early in pregnancy as possible, with the first visit in the first trimester, one in the second trimester and two visits in the third trimester [9]. Despite relatively high coverage of antenatal clinic (ANC) attendance among pregnant women in sub-Saharan Africa, coverage of both interventions across many countries in the region is low [10], limiting achievement of their full potential effectiveness or impact on maternal and neonatal outcomes [11,12]. Case management practices for malaria illness during pregnancy are less well understood and exclusion from national population and facility-based surveys suggests the need for more systematic evaluation through research.

Kenya in East Africa and Mali in West Africa represent two countries with different malaria epidemiology, health systems and socio-economic and cultural settings, both with low coverage of malaria in pregnancy interventions. Kenya adopted the IPTp policy in 1999 and the ITN policy in 2001, and Mali in 2003 and 2006, respectively. According to national survey data for Kenya and Mali available in 2009 when this study was designed, the proportion of women receiving ≥2 doses of IPTp-SP was 4% in both Kenya and Mali, and ITN use the night before the survey was 4% and 49%, respectively [13,14]. Coverage of ≥2 doses of IPTp was substantially lower than the proportion of women making 2 or more ANC visits (84% and 63% in Kenya and Mali respectively) [13,14], indicating substantial missed opportunities to provide IPTp when the pregnant woman was at the ANC. We undertook a systematic examination of the operational, socio-economic and cultural constraints to pregnant women’s access and use of IPTp, LLINs and case management in the diverse settings of these two countries to provide data from which rational strategies aimed at improving coverage could be developed and implemented. We used a combination of health facility and community assessments using quantitative and qualitative methodologies. The household survey, health facility surveys and in-depth interviews with health staff are described elsewhere [15–18]. Here we report the findings of a qualitative study focussing on the community level in Kenya and Mali.

## Methods

### Ethics statement

The study was approved by the ethical committees of the Kenya Medical Research Institute’s (KEMRI) National Ethics Review Committee, Kenya; the Institutional Ethical Committee of the Faculty of Medicine, Pharmacy and Odonto-stomatology, University of Bamako in Mali; the Liverpool School of Tropical Medicine, UK; and the London School of Hygiene and Tropical Medicine, UK; and for the Kenya study, ethics approval was also obtained from the Centres for Disease Control and Prevention, Atlanta, Georgia, USA. All ethics committees approved verbal informed consent to be obtained from study participants as the study procedures posed minimal risk to participants and to avoid the potentially negative influence of written consent on rapport between participants and researchers. With participants’ prior agreement, verbal consent was obtained and recorded prior to the focus group discussions. Pregnant women aged 15–17 years are considered emancipated minors in Kenya and Mali and were consented directly; for adolescents who did not fall into this category, verbal assent of the participant and consent from the guardian was witnessed and recorded. During transcription, any names were replaced with codes to ensure anonymity and digital recordings were deleted once transcription and translation were completed and quality approved.

### Study sites and context

The study was conducted in Kenya and Mali, in 2009 and 2010 respectively, chosen to represent very different African health system, epidemiological, cultural and socioeconomic contexts as part of a larger study to evaluate the barriers to the scale up and use of interventions to control malaria in pregnancy [15–18].

#### Kenya

This work was conducted under KEMRI and CDC’s collaboration in western Kenya. The Kenya study site was Greater Nyando District, Nyanza Province, now divided into three sub-Counties, Nyando, Muhoroni and Nyakach, each managed by a district commissioner. The district has a population of 355,800 projected from the 1999 census, with more than 90% of this population living in rural areas. The district has a total of 40 health facilities of which 24 are government-owned, five by missions, seven privately owned and four community-operated. The most common ethnic group is Luo, who live on the shores of Lake Victoria and the main economic occupation is subsistence agriculture and cultivating cash crops such as rice, sugar cane, sisal and fishing. Malaria in Nyando District is perennial holo-endemic with a parasite prevalence of 8.3% among women of child bearing age (2008 unpublished data, KEMRI/CDC). HIV prevalence among women aged 15–49 years is higher in Nyanza Province compared to all other provinces, 18% compared to a national average of 9% [19].

In line with WHO recommendations on focussed antenatal care (FANC), the Kenya National Guidelines for the diagnosis, treatment and prevention of malaria at the time the study was conducted stated that a package of interventions should be delivered through antenatal care which, in areas of high transmission, includes malaria in pregnancy and prevention of mother to child transmission (PMTCT) alongside other components [20]. These include a free LLIN for all women at first ANC visit and two doses of IPTp-SP given at each ANC visit after quickening, administered under directly observed therapy (DOT), unless SP has been taken in the prior 4 weeks or the woman is HIV-infected and taking daily cotrimoxazole prophylaxis for opportunistic infections. Malaria episodes should be treated with a 7-day course of oral quinine (all trimesters) or artemether-lumefantrine (AL) (2^nd^ & 3^rd^ trimester and 1^st^ trimester if quinine is not available) [21].

#### Mali

The Mali study site was Segou District, Segou Region, which has a total population of 448,552 projected from the 1998 census, with more than 60% of this population living in rural areas. The most common ethnic groups are Bamanan and Sarakole/Soninke, and the main economic occupation is subsistence agriculture. The district has a total of 26 functioning health structures comprising one hospital and one district level health facility (Centre de santé de reference [CSRef]), both of which are government operated and 24 community-owned health centres of which eight are headed by a physician paid by the government and 16 headed by a nurse paid by the community. Malaria in Segou Region is seasonal ranging from holo-endemic in the southern part of the district to meso-endemic in the north. HIV prevalence among women aged 15–49 years is higher in Segou Region compared to the national average, 1.7% vs 1.4% [14].

The health system in Mali has been described elsewhere [17]. The government funds health facilities to district level only, including one hospital and one CSRef. All health facilities below this level are funded by communities themselves, in some cases with assistance from non-governmental organisations. At the time of the study, malaria in pregnancy services provided through ANC included two doses of IPTp administered by DOT between month 4 and 8 (inclusive) gestation, with each dose given at least one month apart, and three doses for women who are HIV positive, in addition to a free LLIN to all women at first ANC visit [22].

### Data collection and participants

The study was undertaken during the rainy season, February-March at the start of the long rainy season in Kenya, and September towards the end of the rainy season in Mali, to capture experience and use of malaria in pregnancy interventions as a current or fairly recent event. Focus group discussions (FGDs) were held in a sample of women of child bearing age (aged 15–49 years) living in two villages from within the study areas. There were three sub-groups within this population, to represent both pregnant and non pregnant women, and adolescents as a high risk group: women who were not pregnant, women who were either currently pregnant or a mother of a child under 1 year, and adolescent girls. FGDs were also held with men living in the selected villages aged 18 years or older. Each sub-group contained 8–12 participants and two FGDs were held per sub-group. Sessions ran for approximately one hour, were conducted in the local languages, Dholuo (Kenya) and Bambara (Mali), and were digitally recorded.

In Kenya, the FGDs took place in Ochoria and Kamahawa villages in Koru and Kakola locations respectively, selected randomly using a sampling frame. The FGDs were conducted by an experienced female social scientist (FA) assisted by a community health worker (note taker). FGD participants were randomly selected from a list of village members provided by the village chief, and field staff visited consecutive names on the list until 12 community members had agreed to participate. In Mali, FGDs were undertaken in Banankoro and Sagni villages, representing urban and rural communities respectively, and participants selected with the help of the village chief and head of the CSCOMs. The FGDs with women were undertaken by three female researchers and an experienced male social scientist (SD) for men. Participants were recruited with the help of community leaders including village chiefs, assistant chiefs and village elders, and the location of the focus groups chosen by the community based on their convenience and proximity to all participants.

FGD guides were developed covering the same topics for both countries. Exploration of the women’s opinions focussed on: experiences during ANC visits including perceptions of services provided at ANC, perceptions of malaria risk and patterns of care seeking, experiences with ITNs, IPTp-SP, and antimalarial drugs for treatment of malaria, and community influences of health seeking behaviour. Exploration of the men’s opinions focussed on their views on the same topics.

### Data analysis

Transcripts were first translated from the local language into English and verified by two of the authors (Kenya: FA; Mali: KK). The translated transcripts were read to get an overview of the themes arising then entered into NVivo10 for data management and analysis. Data from each site were coded separately using a combination of pre-defined themes based on the original research questions and themes that emerged from the data using content analysis. Pre-defined and emerging themes from Kenya and Mali were then compared to explore similarities and differences in the experiences and beliefs concerning access and use of ANC and of malaria in pregnancy interventions, and the views of men in relation to those of women. The FGD findings are used to complement findings from household surveys, and are discussed in relation to data collected from health facilities, in order to strengthen, compare and contrast findings from women and health providers, and from women enrolled in community and health facility settings in each country.

The policy and programme implications of the findings were explored using the WHO health system framework comprising six building blocks: Governance, Human resources, Products and technologies, Service delivery, Information systems and Financing [23].

## Results

A total of 158 participants were recruited into the FGDs, 79 in Kenya and 79 in Mali. The participant numbers by group, village, country and demographics are provided in Table 1. Pre-defined and emergent themes were coded around the five key topics in the topic guide, which were pregnant women’s: access and use of ANC; access and use of case management of malaria in pregnancy; access and use of IPTp; access and use of ITNs; and sources of information, including social and cultural influences.

**Table 1:** Focus group discussion participant demographics.

**FGD group**	**Village**	**Group**	**N**	**Average Age**	**Average Gravidity (range)**	**Marital status**			**Education**		
	**Kenya**					**Single**	**Married**	**Widowed/divorced**	**None**	**Primary**	**Secondary or above**
1	Kakola	WOCBA, not pregnant	10	28	4 (0–12)	0	10	0	1	7	2
1	Koru	WOCBA, not pregnant	10	31	5 (0–9)	1	8	1 W	0	10	0
2	Kakola	Pregnant woman or mother of child <1yr	10	26	3 (1–5)	1	9	0	1	5	4
2	Koru	Pregnant woman or mother of child <1yr	9	23	3 (1–7)	0	9	0	0	6	3
3	Kakola	Adolescent 15–18yr	10	17	1 (0–1)	10	0	0	0	5	5
3	Koru	Adolescent 15–18yr	10	18	2 (1–4)	0	10	0	0	8	2
4	Kakola	Man	10	40	N/A	0	9	1 W	1	8	1
4	Koru	Man	10	38	N/A	0	9	1 D	0	5	5†
	**Mali**										
1	Banankoro	WOCBA, not pregnant	8	45	5 (1–13)	NR	NR	NR	NR	NR	NR
1	Sagni	WOCBA, not pregnant	12	35	5 (0–9)	NR	NR	NR	NR	NR	NR
2	Banankoro	Pregnant woman or mother of child <1yr	10	28	2 (1–6)	NR	NR	NR	NR	NR	NR
2	Sagni	Pregnant woman or mother of child <1yr	12	28	4 (1–7)	NR	NR	NR	NR	NR	NR
3	Banankoro	Adolescent 15–18y	8	17	0	NR	NR	NR	NR	NR	NR
3	Sagni	Adolescent 15–18y	9	17	NR	NR	NR	NR	NR	NR	NR
4	Banankoro	Man	8	49	N/A	NR	NR	NR	NR	NR	NR
4	Sagni	Man	12	40	N/A	NR	NR	NR	NR	NR	NR

Key: WOCBA, women of childbearing age; y, year; NR, not reported

† 2 men had been to college (R3 and R4)

### Access and use of ANC

Factors affecting pregnant women’s access and use of ANC emerging from the data were coded into five main themes: perceptions of the benefits of ANC; experiences at ANC (both positive and negative); health staff corruption and malpractice; poor attitudes of health staff; long waiting times; shame or fear of pregnancy disclosure; and distance and/or cost (Table 2).

**Table 2:** Factors affecting pregnant women’s access and use of ANC.

**Themes**	**KENYA: Sub-themes & Quotations from study participants**	**MALI: Sub-themes & Quotations from study participants**
**Perceptions of benefits of ANC**	**To monitor pregnancy and reduce complications at delivery**	**To monitor pregnancy and reduce complications at delivery**
	*We always go to the clinic so that when my delivery comes in a bad way*, *I cannot be sent away*. (R1: married woman aged 42 years, multiparous, Kakola)	*It is good for the child*. *During the ANC*, *you will know that your child has not a good position and won’t know if you do not go to ANC*. (P7: woman aged 32 years, multiparous, Sagni)
	*Sometimes the baby is lying wrongly so he/she must be checked and also our weight is being measured*. (R4, married woman aged 30 years, multiparous, Kakola)	*Last time with our grandparents*, *there were many deaths of women at delivery due to the absence of ANC*. *Now if the wife does not see her menses for one month and inform her husband*, *her husband sends her to the health centre to know if she is pregnant or if she has another disease*. *If she is pregnant*, *you send her to ANC and you as the husband remind and force her to respect the appointment days*. *Men should always be involved in the health status of his family*. (P8: man aged 55 years, Sagni)
	*I see it good when my wife goes to the clinic because she will have a record that can be used for the case of problem in delivery*. (R6: married man aged 66 years, Kakola).	
	**To test for HIV or other diseases**	**To test for HIV or other diseases**
	*We go to clinic to be taught and be tested of malaria ad HIV when we are pregnant*. *And if found with HIV we are given medicine that can prevent the unborn from getting the disease and also some medicine for malaria you are also given and & vitamins*. (R7: single, adolescent aged 17 years, 1 previous pregnancy, Kakola)	*If you stay at home*, *you will not be informed on certain diseases like fibroma*, *diabetes*, *high blood pressure*, *HIV*, *and many other diseases*. (P3: woman aged 26 years, multiparous, Banankoro)
	*We [men] go with them [our wives] because when the woman goes they ask ‘where is your husband?’ mostly when they want to test the HIV status*. (R7: married man aged 23 years, Kakola)	
	**Other reasons for attending ANC**	**Other reasons for attending ANC**
	*To be given some teachings how they can help the unborn from contacting malaria and how they can prevent other diseases not to affect the unborn*. (R9: single, adolescent aged 18 years, 1 previous pregnancy, Kakola)	*During ANC*, *injections against tetanus are done*. *All this is the advantage of ANC*. (P2 : adolescent aged 17, no previous pregnancies, Banankoro)
	*We always go to get the advice from the doctors and also to get all the vaccinations [tetanus toxoid]*. (R2, married woman aged 35 years, multiparous, Koru)	*They tell you how to take your drug*, *when to come for your appointment*. *The advantages of ANC are enormous*, *the doctors give counselling on the regimens to adopt*, *check your blood pressure*. (P4: woman aged 30 years, multiparous, Banankoro)
**Experiences at ANC (both positive and negative)**	*When you go to the clinic*, *your blood should be tested to determine like HIV status and blood group things like that but sometimes you go but they just test HIV status only*. (R10: single woman aged 20, multiparous, Kakola)	*What discourages me is the behaviour of new nurses*. * *.* *.* *. *The big doctors try and give hand to the new doctors that have no experience*. *Young nurses don’t know the position of the foetus*. *I don’t want the fact that they want us to be examined by the young doctors*, *which is why I don’t want to go there now*. (P9: woman aged 25, multiparous, Banankoro)
	*Sometimes a doctor can just leave a patient even dying in the queue saying that their time for lunch or leaving for home has reached so they may leave a patient dying and this always annoys very much*. *(*R10: single woman aged 20, 1 previous pregnancy, Kakola)	*From the beginning to the end of the pregnancy I did not get sick*. *During my ANC*, *they received me very well and I did not any difficulty during the delivery*. (P5: adolescent aged 16 years, no previous pregnancies, Banankoro)
		*Nobody goes to health centre because of pleasure*! (P7: woman aged 28 years, multiparous, Banankoro)
**Health staff corruption and malpractice**	*You can go and make a line and see that you are about to reach*, *but when their relative or some rich person had come*, *they will take them in the front and give them treatment*. * *.* *.* *.*they are always corrupt*.*(*R1, single, adolescent aged 18 years, 1 previous pregnancy, Kakola)	*The reception must be very attractive at the health centre*. *Sometimes the doctor comes to the health centre and do not receive people for consultation*. *They transfer their duties to the nurse or midwife*, *while this [person] cannot do the work*. (P7: adolescent aged 16, no previous pregnancies, Banankoro)
	*If you go to private hospitals you can be forced to be tested but this should only be your wish so as to know your status*. (R8: married, adolescent aged 17 years, 3 previous pregnancies all alive, Koru).	
**Poor attitudes of health staff**	*Sometimes they can call your name twice and you don’t hear*, *so when you go inside they will start harassing you saying we didn’t send you*! *We were not there*! *So if you go for the first time and they handle you like that*, *then you will not go for the second time like for me they handled me that way but I left and even left for them the book and only waited for six months then went to Kisumu*. (R9: single, adolescent aged 18 years, 1 previous pregnancy, Kakola)	*Doctors must be kind with us*. *There are certain doctors who are not very kind and shout at pregnant women and when you go for delivery some insult you*. (P8: woman aged 25 years, multiparous, Banankoro)
		*What discourage people is that they are neglected*, *the manner the health receive patient is not good as it does not create confidence. .... That is the reason why people don’t want to go to the health centre*, *the doctors are not accessible*. (P1: man aged 71, Banankoro)
**Long waiting times**	. * *.* *. *if it reaches twelve o’clock*, *they will go for lunch and tell you to wait until two pm*.*(*R1, single, adolescent aged 18 years, 1 previous pregnancy, Kakola)	*Sometimes you can spend one hour or more without seeing the doctors*. *That is the reason why people don’t want to go to the health centre*, *the doctors are not accessible*. *But*, *even if they are not good in receiving patient*, *you are obliged to go there and to be very patient*. (P1: man aged 71, Banankoro)
**Shame or fear of pregnancy disclosure**	*It is feeling ashamed*, *because sometimes you are going to the clinic at the same time your mother is also going now you feel ashamed*. (R9: single, adolescent aged 18 years, 1 previous pregnancy, alive, Kakola)	*The husband asks [you] to hide the pregnancy because pregnancy is always a psychological problem*. (P8 : woman aged 27 years, multiparous, Sagni)
	*It is feeling ashamed because sometimes you have not tried so you can ask someone*, *what do they normally ask*, *they ask if you are married and other things so it is only feeling ashamed*. (R4, single, adolescent aged 17 years, no previous pregnancies, Kakola)	*Because during pregnancy*, *women get many discomfort*. *For each discomfort you can’t go to the health centre otherwise your husband will not be happy on you*. *They say that always to pose financial problems*. (P10 : woman aged 28 years, 4 previous pregnancies, Sagni)
	*Some fear that they will be seen while pregnant*. (R4: single, adolescent aged 17 years, no previous pregnancies, Kakola)	
**Distance**	*The hospitals are far and sometimes you are in the interior so when you go*, *you can reach when tired and the nurses will not treat well because you also don’t have money*. (R4, single, adolescent aged 17 years, no previous pregnancies, Kakola)	*For example if the baby is not in a good position*, *because here we don’t have the means of quick transportation and it is very frequent that people are killed because of that*. (P2: woman aged 23 years, multiparous, Sagni)
**Cost**	*People always go to the TBAs just because of poverty and lack of money but they are not good* (R6: married man aged 66 years, Kakola).	*If you don’t have money and your husband also*, *you are obliged to stay at home*. (P6: woman aged 36 years, multiparous, Banankoro)
	*I went and they asked me if I have ever done it [ANC] and I said no it’s when I want to start and they needed money and I didn’t have money so they started quarrelling why I didn’t have money and it is done with money*. *(R10*: single, adolescent aged 17 years, 1 pregnancy, *Kakola)*	

#### Perceptions of benefits of ANC

The key motivation for women to attend ANC in both Kenya and Mali was to have their pregnancy monitored and managed in the hope this would lead to a healthy pregnancy and, ultimately, reduce the risk of complications at delivery. Knowing the position of the baby was important and having the baby in the correct position for delivery was seen to be critical for avoiding problems at delivery. Women who didn’t attend ANC at least once were said to risk being sent away by midwives if they came with complications at delivery. During the FGD at Kakola village in Kenya, a 66 year old married man described the benefit of his wife attending clinic was that she would have a record of her treatment and care, which would be useful in case there were problems at delivery. Men in Mali were also aware of the dangers of women not obtaining regular antenatal care, and referred to the ‘past’ when many women died during delivery due to the absence of adequate antenatal care services.

Another key element of attending antenatal clinics referred to by women was to have tests for HIV and other diseases, including malaria, so that ‘tablets against malaria’ could be taken to safeguard the health status of both her and the unborn child. Preventing the onset of malaria was said to save money compared to treatment of the illness once it had progressed. Knowing one’s HIV status was more commonly mentioned by women in Kenya, where men were said to sometimes accompany their wives as the clinics encouraged partner testing for HIV. Receiving counselling on disease prevention and professional advice on the use of safe regimens during pregnancy and tetanus immunisation were additional reasons cited for attending ANC. Monitoring and information to control high blood pressure and receiving iron was mentioned by women in Mali but not in Kenya. Testing for blood group was mentioned in Kenya but not in Mali.

#### Experiences of ANC service quality (positive and negative)

Experiences of ANC were predominantly negative, as remarked by a 28 year old, multiparous woman in Banankoro village in Mali ‘*Nobody goes to health centre for pleasure’*, although there were some positive experiences too. There were instances where women didn’t get all the services they would have liked, as observed by a single, multiparous woman aged 20 in Kakola village, Kenya, who complained that although they are supposed to be given a blood group test, sometimes they were only given an HIV test. Some adolescent women were fearful of going to clinics because they would be tested for HIV, whereas others were afraid of taking ‘medicines’ or of being pricked for a blood test. Although health staff at ANC were generally considered to be well trained and experienced, there were examples in Mali where mothers reported that young nurses were unable to do essential procedures such as determining the position of the foetus, and some women stated that they avoided attending particular clinics for that reason.

#### Health staff corruption or malpractice

Corruption among staff was identified as a problem in Kenya, with staff demanding bribes before agreeing to see patients and showing favouritism toward ‘*rich’* clients. Private sector providers in Kenya reportedly refused to see patients unwilling to have HIV tests. In Mali, doctors were reported to sometimes refer their duties to the nurse or midwife, who was unable to do the work of a doctor.

#### Poor attitudes of health staff

Women in both countries complained about the rude and sometimes bullying behaviour of health staff, both doctors and nurses. Women in Mali claimed they were frequently shouted at and insulted, and were often neglected. Neglect was also mentioned in Kenya, where a single, 20 year old woman in Kakola village with one previous pregnancy woman said patients were left in a queue whilst the doctor went for lunch. Another woman in Kenya reported delaying ANC until the 6^th^ month of pregnancy due to the rudeness of the nurses. Women in Mali reported lack of staff morality and ‘poor reception’ at health facilities as reasons for avoiding certain health facilities.

#### Long waiting times

Women and men in both Kenya and Mali complained of long waiting times at clinics. In Kenya, women said they were made to wait for two hours between 12–2pm whilst the nurses ‘went for lunch’. In Mali, long waiting times were said to be a disincentive for patients attending health centres.

#### Shame or fear of pregnancy disclosure

Shame and fear of pregnancy disclosure was a common theme in both countries, but of very different natures. In Kenya, shame was associated with an adolescent being pregnant out of wedlock, or being pregnant at the same time as their mother so that they had to attend the clinic together. In Mali, women were conscious of not disclosing ailments associated with pregnancy to their husbands; husbands perceived ailments to be too frequent causing them irritation due to the costs associated with seeking care, for which the husband is responsible. A 27 year old multiparous woman in Sagni village said she did not like to disclose her pregnancy because her husband regarded pregnancy as a ‘psychological problem’.

#### Distance and/or Cost

Distance from a hospital or health facility was cited as a problem mainly because of lack of transportation, especially in Mali where lack of transport was said to result in the death of many women, for example when the baby was not in the correct position at delivery. Lack of money was said to be the main barrier to attending ANC. In Kenya, distance and lack of money went hand in hand, and money was seen as an essential requirement to receiving antenatal services. There were examples in both countries where women were offered a credit system. In Mali, this involved a temporary credit, applied by the health facility, whilst women found the money to reimburse the clinic. In Kenya, a district hospital was said to sponsor patients who could not afford to pay for inpatient care.

### Access and use of treatment for malaria

Factors affecting pregnant women’s treatment seeking for malaria emerging from the data were categorised into six main themes: perceptions of illnesses and diagnosis; perceptions of treatment drugs; experience of treatment drugs; pregnancy disclosure; cost (affecting source and type of treatment); and perceptions of different providers and other factors affecting source of treatment (Table 3).

**Table 3:** Factors affecting pregnant women’s practices for the treatment of malaria.

**Themes**	**KENYA: Sub-themes & Quotations from study participants**	**MALI: Sub-themes & Quotations from study participants**
**Perceptions of illnesses and diagnosis**	*I didn’t know that it was malaria so I used different medicine not knowing that it is malaria*. *I thought that I was preventing some diseases like pneumonia so whenever I feel any cold*, *Panadol is the only option that I use and when it didn’t get over*, *I went to the hospital and was tested and found out that it was malaria*. (R9, single, adolescent aged 18 years, 1 previous pregnancy who is alive, Kakola)	*Pregnant women suffer the most* [from malaria] *because they confuse the [malaria] symptoms with pregnancy symptoms so that you don’t know that you have ‘sumaya’ [malaria] and this give opportunity to infection to stay longer in your body*, *and you discover very late that your illness has advanced*. (P4, woman aged 20 years, no previous pregnancies, Sagni)
	*When I am pregnant and I get attacked with malaria*, *I must go to the hospital so that the doctor can test me because if I go to the chemist and I can be given drug that harm my baby that I carry*. (R1, married women aged 28 years, multiparous, Kakola)	*Some have ‘sumaya’ [malaria] but they think that it is the beginning of pregnancy*. *So if they explain to a health worker*, *they can get more clarifications*. *If it is pregnancy*, *they provide counselling and ask you to sleep under bednet*. (P8: adolescent aged 18 years, no previous pregnancies, Sagni)
	*But what I fear is the syringe though I can swallow even a pale of medicine*. *But when I go to the hospital and a time for lab test comes*, *I will not go*. (R9: single, adolescent aged 18 years, 1 previous pregnancy, Kakola)	*The abused use of traditional plant can cause other diseases*. *It is good that you get to the health centre for diagnosis before you get any treatment*. (P5: woman aged 40 years, multiparous, Banankoro)
		‘*KONO*’ [cerebral malaria] *which means that the body becomes hot and stiffness and we go to the health centre*. (P5: woman aged 40 years, multiparous, Banankoro)
**Perceptions of treatment drugs**	**Effectiveness**	**Effectiveness**
	*In the past we were using drugs like Algon and Fansidar but they were very effective but the Fansidar of nowadays are not effective*, *it is like they have added some chemicals that is why it is ineffective*. (R10, married man aged 20 years, Kakola)	*We are treated using traditional medicine and modern medications*. *But modern medications are rapid [treated more quickly]*. (P8: adolescent aged 18 years, no previous pregnancies, Sagni)
	*Drugs used nowadays are more effective than the past ones*. (R3, married man aged 23 years, Kakola)	**Confusion over use**
	*These medicines only work if we use them accordingly*. (R 8, married, adolescent aged 17 years, multiparous, Koru)	*Both prevention and treatment*. *It [ACT] also contain vitamin inside*. (P6: woman aged 36 years, multiparous, Banankoro)
	**Safety**	‘**Habit’ of ‘modern medicine’**
	*There are some bad effects because some have not undergone a study and we cannot tell the dosage*, *some are not for malaria though they are indicated but they are for some companies which want to make profit out of us and these medicines are not tested that they can treat malaria*. (Respondent 7, married man aged 36 years, Koru)	*We used to give herb to our wife*, *but now we bring her to the health centre*. *…*. *Since the day of birth the child received modern medication and 15 days after he receives another modern medication so that the blood and the body of the child take habit of the drug and finally the traditional plant cannot help the child for treatment*. *So it is good to get to the health centre*. *You can do a week to treat malaria with traditional plants without any success*, *but with three days of modern treatment you get success*. (P10: man aged 41 years, Sagni)
	*You cannot take Coartem when you are pregnant because it is powerful l like Fansidar*. (R2, married woman aged 31 years, multiparous, Kakola)	
	*The past anti-malarial drugs like quinine are not good with expectant mothers*. (R1, married, adolescent aged 18 years, multiparous, Koru)	
**Experience of treatment drugs**	**Side effects**	**Side effects**
	*I have heard about it [quinine] and I have used it but after that*, *I felt like I was deaf*. (R4, married, adolescent aged 17 years, multiparous, Koru). *I used it but felt as if my ears were blocked*. (R8, married, adolescent aged 17 years multiparous, Koru)	*Me I took it and as side effect it gives buzzing in ears and giddiness*, *myself I wanted to refuse to take the remainder*, *but it fights well against ‘sumaya’* [malaria]. (P4, woman aged 20 years, no previous pregnancies, Sagni)
	*I had malaria and that is why I used quinine and I didn’t like it because I was scratching my body*. (R5, married pregnant woman aged 20 years, first pregnancy, Koru)	*I take it [quinine] when I eat enough to avoid adverse events*. (P1: woman aged 48 years, 1 previous pregnancy, Banankoro)
	**Effectiveness**	**Effectiveness**
	*If I swallowed the ones used in the past I don’t see any change but the new ones like Coartem if I swallow I still continue feeling headache but after a day I feel okay*. (R2, married woman aged 35, multiparous, Koru)	*We found that quinine is not working anymore and this disturbs us a lot*. *Before there were many types presentation of quinine injection*: *0*.*30; 0*.*40; 0*.*60*. *Even three doses of 0*.*60 is no more enough for treatment*. *Now they recommend 0*.*80 that is not also enough*. *We used quinine injection and traditional herbs*. *But we are still confused*. (P5: woman aged 49 years, multiparous, Banankoro)
**Pregnancy disclosure**	**Moderator: When you were pregnant even though others did not know, do you think it was better that you tell sister who works in the clinic for women, drug sellers, the person you buy drug from in the shop your pregnancy status?**	**Moderator: Before you take drug during your last/current pregnancy, does the prescriber ask to see if you are pregnant before you take the drug?**
	*I think that it is a good to tell the one who is treating you so that he doesn’t give you drugs that can make you miscarry*. (R7,married woman aged 28 years, multiparous, Koru)	*I had a ‘furoncle’* [boil], *the doctor asked me if I am pregnant*, *I said yes and he changed my treatment*. (P10: woman aged 28 years, multiparous, Sagni)
	*I had to tell them so as to give me the right medicine which is good with an expectant mother*. (R1, married, adolescent aged 18 years, multiparous, Koru)	*He asked me if I have seen my menses*. *I responded no*, *he asked me whether I suffer and I informed him*. *He gave me a prescription*. (P3: woman aged 27 years, multiparous, Sagni)
	*Pregnancy that is starting*, *there is nobody who can ask you because it is not visible*. (R1, married woman aged 42 years, multiparous, Kakola)	*No*, *my pregnancy has 4 months of pregnancy without people know that I am pregnant*. (P5: adolescent aged 18, no previous pregnancies, Banankoro)
	*I cannot tell them because I don’t want them to know that I am pregnant*. R6: married women aged 34 years, multiparous, Kakola	
**Cost of drugs (affecting source of treatment)**	*I use Fansidar because it is the cheapest but Coartem is very expensive*. (R10: married man aged 20 years, Kakola).	*The publicity on the free provision of ACTs poses many problems*. *It is right that on the prescriptions the ACTs are free but the other partner drugs are not free*, *and people cannot understand that*. *For me*, *the publicity must say that only the ACTs are free not the other partner drugs on the prescription*. *This gratuitous poses a lot of confusions mainly with the relais people*. *People said that*, *the doctors and the relais are the one who take advantage (sell) of this drug for themselves*. *We explained that the other drugs on the prescription are for other disease*. *The publicity gives the impression that we do not spend any money for the treatment of malaria*. (P10: man aged 41 years, Sagni)
		*There is a need to reduce the fees of ‘les antipaludiques’ [antimalarials]*. *Drugs are expensive*. *That is why people are referring to the traditional plants*. *With 100 CFA* [USD 0.2], *you can buy plants for treatment*. *But in the health centre*, *you pay more and you are recovered*. *People prefer to stay home*. (P5 : woman aged 37 years, multiparous, Banankoro)
		*We made our ANV and EPI correctly and the doctors refused to provide moustiquaire imprégnée d ‘insecticide [impregnated bed net]*. *Later when we get malaria they provide drug order to buy drug*. *That is why use traditional plant at home*. (P4 : woman aged 30 years, multiparous, Banankoro)
**Factors affecting source of treatment and perceptions of different providers (*in addition to cost*, *above*)**	**Lack of drugs**	**Complex inter-play of several factors**
	*The challenge is where we go to get medical services because we always don’t get drugs but are just being told to buy so sometimes we find no need on going to the hospital other than just buying drugs from the pharmacies*. (R5: married woman aged 35 years, multiparous, Kakola)	*I go to the health centre to verify that I have ‘sumaya’ [malaria]*. *If it is ‘sumaya’ I use traditional medicine because I am afraid of the costs of the modern medicines*. *We do not have enough money to face to the drug order*. *There is a plant called ‘TINKILOLA’ when I use concoction* [boiling herbs in water] *for that plant with a glass of water*. *This treatment is good*. (R6: man aged 63 years, Banankoro)
	**Competence**	**Competence**
	*Yes that’s how we do because some of us fear medicine therefore is recommended to go to the hospital so that you can be told how to use such medicine*. (R10: married, adolescent aged 18 years, 1 previous pregnancy and currently pregnant, Koru)	*To know about what disease you suffer from*. *I spent 6 days using traditional medicine because I was ill*. *I thought that I have a cold but my parents ask me to go to the health centre*. *When I went there*, *the doctors asked me if I sleep under impregnated bednet and I responded yes*. *They said that I have malaria*. (P5: adolescent aged 18, no previous pregnancies, Banankoro)
	*When I am pregnant but it forces me to go to the hospital depending on my pregnancy status*. *I cannot buy medicine anywhere*. (R4:married woman aged 21 years, multiparous, Koru)	*Pregnant women are like infants*, *we bring them to the health centre*, *with them we don’t use traditional medicine*. (P2: man aged 55 years, Banankoro)
	**Convenience**	
	*I first tried with the ones at the shop and when it didn’t stop*, *I went to the hospital and I was tested and found out that it was malaria then I was injected*. (R10, single, adolescent aged 17 years, multiparous, Kakola)	
	*I send her [wife] to a private hospital to get fast treatment*. (R6: married man aged 30 years, Koru)	
	*It is now 2 weeks since I got infected with malaria*, *when it started me it started with body hotness and my head was also aching and I bought the drugs from the chemist and I took and I saw it was just persisting*. *So I got some money and went to the hospital and I was tested and I was given drugs and it got over*. (R4, married woman aged 30, multiparous, Kakola)	
	**Fear**	**Fear**
	*There are some women who are used to local medicine and there are those that don’t go for test and they can be afraid going to the clinic*. (R7, married man aged 36 years, Koru)	*If you don’t know somebody there*, *you will not have good reception at the health centre*. *I don’t like injections*. (P3 :adolescent aged 18, no previous pregnancies, Banankoro)

#### Perceptions of illnesses and diagnosis

Malaria in pregnancy was widely considered to be dangerous and a serious threat to both the pregnant women and their unborn children, causing ‘weakening the blood’, headache, vomiting, hot body and dizziness. It was said to result in small babies, babies born with malaria, abortion, and death. Malaria was described as a hidden illness that can kill fast, and could easily be confused with pregnancy symptoms (Mali) or with other diseases such as pneumonia (Kenya and Mali). Some women claimed that malaria symptoms could be confused with pregnancy so that sometimes treatment was sought very late, after the infection was well established.

These factors combined suggest an importance placed by women in both countries on suspected cases requiring diagnoses at a health centre or hospital, before seeking treatment. In Mali, severity of the illness was also a consideration for going to a health centre, for example when pregnant women or infants had severe malaria, known locally as ‘*kono’* (used also to mean severe or cerebral malaria).

#### Perceptions of treatment drugs

Women and men in both countries had many preconceptions about the different antimalarials or herbal medicines available for the treatment of malaria, mostly on their safety or efficacy for use in pregnancy. Women and men in Kenya correctly observed that ‘*Fansidar’* (SP) was no longer effective for treatment of malaria, and the men explained that some chemicals had been added or that the doses were incorrect. The newer drugs were widely perceived to be more effective than the older drugs in both countries. Kenyan women were concerned about using drugs correctly (as prescribed) and about safety, and Coartem (AL) and quinine were said to be too powerful to be used in for treatment in pregnancy. Drugs obtained from chemists were said to potentially harm the baby and the preference was to obtain treatment from a trained provider at a health facility following diagnostic confirmation.

In Mali, both herbal remedies and bio-medicines were reportedly used to treat malaria in pregnancy, and herbal remedies used if women could not afford bio-medicines. Bio-medicines were seen to be more effective than herbal remedies, with a more rapid mode of action, but there was some confusion over the application of some drugs such as ACTs, which were thought to be used for both treatment and prevention. In Mali, the misuse of traditional plants for treatment was seen to cause problems, and if someone had received only bio-medicines from birth, traditional herbal remedies were said to be no longer be effective in adulthood; we labelled this sub-theme the ‘*habit’* of bio-medicines.

#### Experience of treatment drugs

Women in both countries complained of side effects from some of the antimalarials they had used; quinine was said to cause deafness and scratching or itchiness (Kenya) or buzzing in the ears and dizziness (Mali). One woman in Mali observed that she could avoid adverse events if she ate enough before taking quinine. There were mixed views among women in both countries as to the effectiveness of quinine, being either very effective at fighting malaria or to no longer work at any dose of quinine injection (300 mg, 400 mg, 600 mg, and 800 mg)(Mali). Similarly, SP was said to cause nausea and to no longer work for treatment in Mali.

#### Pregnancy disclosure to prescribers

Disclosing one’s pregnancy to the person prescribing treatment was recognised as important by most women in Kenya and Mali, to avoid being given harmful drugs that may cause miscarriage and because, as early pregnancy was not visible, providers would not always think to ask a woman whether or not she is pregnant. However not all women were willing to disclose their pregnancy status, as described above in response to questions about ANC access.

#### Cost (affecting source and type of treatment)

Affordability was the main reason for women not to seek treatment at a formal health facility or to choose one type of medication over another, especially in Mali where people without money resorted to herbal medicines. Drugs on the whole were considered too expensive, and men in Mali complained that the publicity about ACTs was confusing; although the ACTs prescribed to pregnant women were free, the partner drugs were not free. Doctors and community health workers (known as *relais*) were said to take advantage of this situation by selling the partners drugs themselves.

#### Perceptions of different providers and other factors affecting source of treatment

The factors affecting women’s choice of treatment source represent a complex inter-play of several factors, including drug availability, competence of health staff, convenience and fear of a particular provider. In Kenya, although formal health services were the preferred choice due to competence of the providers, drug stock-outs meant that women often ended up buying the drugs from pharmacies, and surmised that they may as well have gone directly to the pharmacy. Convenience was also an important consideration, resulting in local shops and chemists being used as a first port of call, resorting to a health facility only if the condition did not improve. In Mali, the situation was very different; women who could afford to showed a preference for going to a health centre to get tested for a diagnosis and to be prescribed bio-medicines. The decision of where they go was also influenced by the type of reception they thought they would receive at the health centre.

### Access and use of IPTp

Factors affecting pregnant women’s access and use of IPTp based on the data were categorised into five main themes: effectiveness; application(s); dose regimen; safety; and side effects (Table 4).

**Table 4:** Factors affecting pregnant women’s practices for the prevention of malaria: IPTp.

**Themes**	**KENYA: Sub-themes & Quotations from study participants**	**MALI: Sub-themes & Quotations from study participants**
**Effectiveness**	*Fansidar was for malaria but malaria of this time it cannot treat*. (R10: single woman aged 20 years, 1 previous pregnancy, Kakola)	*No*, *it [SP] is given free*. *But it gives a little malaise*, *dizziness*. *At delivery I got ‘le palu’ [malaria]*. (P1 : woman aged 26 years, first pregnancy, Banankoro)
		*It gave good health as well as my baby*. *I did not get sick and my baby also from the beginning to the end of the pregnancy I never get sick*, *I never get vomit*. (P5: adolescent aged 18, no previous pregnancies, Banankoro)
**Safety**	*It is not good because it is powerful and it can make you miscarry*. (R7 married woman aged 32 years, multiparous, Koru)	
	*It [Fansidar] is not okay because it can affect the baby*.(R3, married woman aged 20 years, no previous pregnancies, Kakola)	
**Side effects and eating**	*I used it but it almost turned me mad; I was feeling body rashes and the headache also advanced and was serious*. (R4: married woman aged 20, multiparous, Kakola)	*I got it also*, *and I got recovered*. *I got it in the health centre*. *I used the treatment without any adverse event*. (P6 : woman aged 23 years, multiparous, Sagni)
		*It [SP] made me sick before I get recovery because I took it when I was hungry and I get dizziness*. (F8: adolescent aged 15, no previous pregnancies, Banankoro)
		*I got it [SP] at the CSREF*, *they ask to eat a lot before you take it*. *I took mine at home*. *It gave me dizziness*. (P2: woman aged 21 years, first pregnancy, Banankoro)
**Use for treatment and/or prevention**	*When I went to the clinic I was given fansidar to help me prevent malaria and I did not pay any cash*. (R8: married, adolescent aged 17 years, multiparous, Koru).	*Before*, *we used chloroquine*, *our parents used it for prevention*. *My father was physician*, *he used chloroquine for prevention*. *He used to give us chloroquine regularly*. *But with new drug*, *we are obliged to take them*. *I take fansidar for prevention*. (P1 : woman aged 48 years, 1 previous pregnancy, Banankoro)
	*It helps prevent malaria and I was told to go and buy in the chemist*. (R4: married, adolescent aged 17 years, multiparous, Koru)	*It [SP] is also used to treat ‘le paludisme’ [malaria]*. *I got it during the CPN [ANC]*. (P4 : woman aged 36 years, 7 previous pregnancies, Sagni)
**Number of tablets and doses**		*When you take it*, *it is not allowed to take another medicine for a week*. *I took it at home*. (P5 : woman aged 37 years, 6 previous pregnancies, Banankoro)
		*Yes*, *I even have the habit of that*. *They say that 3 tablets have to be taken together*. *The week that I used it*, *I did not use any other drug*. *I took the second dose while I am half of my pregnancy*. *In total I took two doses*. (P5: adolescent aged 18, no previous pregnancies, Banankoro)

#### Effectiveness and Application(s)

The views of women in Mali of the effectiveness of IPTp-SP were inconsistent, with women stating that IPTp-SP had been both effective and ineffective at preventing malaria. Although women in Kenya and Mali correctly identified SP for prevention, several women in Mali claimed that SP was still being used to treat malaria.

#### Dose regimen, Safety and Side effects

Although not explicitly requested, an adolescent woman aged 18 years in Banankoro village in Mali was able to state the correct administration of IPTp-SP (3 tablets taken together and a second dose half way through her pregnancy), and that she took no other drugs for a week after taking it. Women at the CSRef were asked to ‘eat a lot’ before they took IPTp, suggesting they were not given it by DOT. Women in Mali had conflicting experiences of side effects (malaise and dizziness) or no side effects, and linked the vomiting associated with taking SP with taking the drug when hungry. A Kenyan woman claimed that SP gave her body rashes and a severe headache. Several women from both villages in Kenya thought that SP was too powerful for use in pregnancy, and that it could harm the baby or cause miscarriage.

### Access and use of ITNs

Factors affecting pregnant women’s access and use of ITNs were categorised into five main themes: sources of nets; stock outs; unofficial practices among health providers; effectiveness; and seasonal use or other factors affecting use (Table 5).

**Table 5:** Factors affecting pregnant women’s practices for the prevention of malaria: ITNs.

**Themes**	**KENYA: Sub-themes & Quotations from study participants**	**MALI: Sub-themes & Quotations from study participants**
**Effectiveness**	*I always use net and wash it after the level of power tab has gone down*. (R5: married man aged 33 years, Koru)	*To protect against malaria*. *Me and my children sleep under a net*. *If it becomes dirty*, *I impregnate it using an insecticide called ‘Bloc'*. (P8, woman aged 42 years, multiparous, Sagni)
**Stock outs**	*I went to the hospital and I found that the nets were over*. (R6: married woman aged 30 years, multiparous, Koru)	*They said that it is finished*, *while in fact after one ANC and EPI*, *they must provide you an impregnated bed net*. *But any time if you go*, *it is finished*. (P4: woman aged 30 years, multiparous, Banankoro)
**Free or not; from ANC or other**	*I bought the first net but when the campaign for malaria came I was given some from the people from that campaign*. (R6: married man aged 66 years, Kakola)	*If you spend more time before you get pregnant*, *you use insecticide used for your moustiquaire imprégnée d ‘insecticide*. *You can also get it during the vaccination (immunization) of children less than 5*. *Here in our health centre we don’t buy it*. (P3; woman aged 42 years, multiparous, Sagni)
	*I took the baby to the clinic and I was given net free*. (R7: married adolescent aged 18 years, 3 previous pregnancies, 2 children, Koru)	*During EPI vaccination at the health centre*, *I got it and I use it during the night to be protected against mosquitoes and small insects*. *Since I start to use it I sleep very well*. (P1: adolescent aged 16, no previous pregnancies, Sagni)
	*I have one which I was given free when I went to the clinic while pregnant*. (R1: married adolescent aged 18 years, multiparous, Koru)	*I got mine during the EPI vaccination of my brother and they prescribe him also drugs*. (P4: adolescent aged 16, no previous pregnancies, Sagni)
	*I went to the hospital and I was given free of charge*. (R4: married, adolescent aged 17 years, multiparous, Koru)	*You do a number of visits to ANC and they provide to you*. (P10 : woman aged 28 years, multiparous, Sagni)
		*For the first time at the ANC*, *and when the child got 9 months if you come for EPI they provide you mosquito net*. (P5, woman aged 41 years, multiparous, Sagni)
**Seasonal use or other factors affecting use**	*Even though my wife use net but not always because you might think that the children are under net but not and when it’s rainy season*, *there might be some stagnant water where mosquitoes breed*. (R2: married man aged 50 years, Koru)	*Sometimes I am lazy to attach my bednet*. *If my sister doesn’t attach it*, *I just sleep like that*. (P2: adolescent aged 17, no previous pregnancies, Banankoro)
	*I can’t lie that we always use net because a times there are no mosquitoes but if it is rainy season*, *we use nets especially for the young one to prevent mosquitoes*. (R1: married man aged 44 years, Koru)	*If we don’t sleep under bednet*, *then we won’t be able to sleep [too many mosquitoes]*. (P10 : woman aged 28 years, multiparous, Sagni)
		*In this period [rainy season] all people sleep under a bednet*. (P7: woman aged 30 years, multiparous, Sagni)
**Favouritism and providers selling free nets to clients**	*Sometimes you may go to the hospital that today I must ask the nurse for a net and you may find the nurse hungry that she informs you that nets we are selling*! (R4: married woman aged 20, multiparous, Kakola)	*I have faith in one doctor from [name health centre] because I did all my ANC*, *but when bednet arrived she said that it is not for us*, *but for others*. *We were 6 people in that situation*. *We use taxi and other means of transport to come to the health centre and they refuse to provide us bednet*. *The doctors sell impregnated bed net*. (P3 : woman aged 26 years, multiparous, Banankoro)
		*It is segregation*, *favouritism*, *and all kind of things*. *For example*, *if they received 40 bednets*, *the doctors give to 5–6 people who did not visit the health centre*. *If we get there*, *they said that it is finished*. (P4 : woman aged 30 years, multiparous, Banankoro)

#### Sources of nets, the sale of free nets/favouritism and stock-outs

Women in Kenya and Mali obtained their ITNs from a variety of sources though predominantly from ANC or hospital, child health clinics and EPI, mainly (though not always) provided free-of-charge. Other sources of nets in Kenya were campaigns or shops, and in Mali, the pharmacy or from their parents (adolescents). Although most women said ITNs were given to them free at ANC, one woman in Kenya complained she was charged. In Mali, several women complained that they did not always receive their free ITN, and that doctors gave the ITNs to other women who had not attended ANC, or simply that the ITNs were not meant for them. There were conflicting reports of when they were given the ITN at ANC, with women in the same village in Mali claiming different circumstances; one woman claimed women should get an ITN after ‘a number of’ ANC visits, and another that you got a net at your first ANC visit, and another when your child got their immunizations at 9 months. Women in both Kenya and Mali reported stock-outs of nets at ANC, hospital and EPI clinics.

#### Effectiveness, Seasonal use or other factors affecting use

ITNs were understood to have an ‘effect’ which waned when the nets got too dirty so women and men reported that they ‘refreshed’ the insecticide with *Powertab* (Kenya) or ‘re-impregnated’ it with *Bloc* (Mali).

Although women claimed they almost always used their ITNs, men in Kenya stated that their wives did not always use them, especially when there were no mosquitoes. In Mali, women reported that they had to sleep under a mosquito net or else they wouldn’t be able to sleep due to mosquito nuisance, however another woman alluded to seasonal use. Use among adolescents appeared to be less of a habit; an adolescent aged 17 years with no previous pregnancies in Banankoro village claimed that she was too lazy to attach the net and if her sister didn’t hang it, she would not use it.

### Household decision making on health seeking

Women in both countries stated that husbands played an important role in decisions over care seeking among pregnant women, although the majority of women admitted that if their husbands were not available, amenable or able to pay for health care, they would go regardless. Women generally asked their husband, as chief of the family, permission before they sought health care, mainly because the husband was also expected to pay for this care. In Mali, husbands were said to force their wives to take any medication prescribed by doctors, including antimalarials, and parents or in-laws also provided encouragement or advice on health seeking.

Other important influencing agents were elders (Kenya) or village chiefs (Mali), midwives and doctors, and *relais* (Mali) or older members of the community or neighbours (Kenya). In Kenya, some churches were said to forbid believers from going to hospital. Radio and TV were also said to influence health care seeking for ITNs and drugs in Kenya and Mali, respectively.

## Discussion

The FGDs with different groups of women of childbearing age and men in Kenya and Mali provides useful information that can be used by the malaria and reproductive health programmes in both countries to inform strategies to improve the delivery and uptake of IPTp, ITNs and case management of malaria in pregnancy. The qualitative data supports many of the quantitative findings from both the household surveys [17,18] and health facility surveys [15,16] that were undertaken in parallel to this study with regard to ANC access and coverage of IPTp and ITNs.

Women recognised the benefits of ANC and were eager to attend ANC despite some negative perceptions and inconvenience so as to ensure a safe pregnancy and to avoid complications at delivery, or to facilitate admission without problems in case complications at delivery, a finding consistent with another study conducted in Kenya, Ghana and Malawi [24] and is consistent with high rates of ANC use in another study in a semi-urban area of Mali [25]. Particular mention was made of wanting to have their pregnancy monitored and getting the position of the baby checked. The professional advice and testing services of professional health staff were valued and actively sought, in preference to shop bought medicines and herbal remedies; this trust in health providers has been observed elsewhere [26]. These factors serve to promote women’s demand for ANC services; however poor quality services and poor attitudes amongst health staff undermine this demand and deter women from using ANC services more frequently. This was illustrated in the household surveys in Kenya and Mali; although 87% and 81% of women made at least one ANC visit respectively, only 33% and 42% made 4 or more visits respectively [17,18], the drop in ANC attendance was particularly marked in Kenya.

Poor health provider practices were a concern in both countries, and specifically poor behaviour among health providers in Kenya and poor quality services and reception in Mali. These issues are not new, and add to the body of evidence calling for improvements in the quality of antenatal care in general [24,27]. The cost of accessing antenatal care was perceived as the biggest barrier to accessing ANC in both Kenya and Mali, as described in a review of access to ANC [27]. Although many women make at least one ANC visit, the cumulative costs of repeat ANC visits over the course of a pregnancy, together with poor quality services, reduce the likelihood of women making repeat visits. Kenya policy historically has been to provide free ANC services (palpation, family planning, HIV testing, IPTp, ITNs and tetanus toxoid), although tests for the ANC profile (urinalysis, Hb, syphilis and blood grouping) are routinely charged. In Mali, charges are levied for most services apart from ITNs.

Individual barriers to ANC access, such as fear of blood testing for HIV, injections or taking certain drugs in pregnancy, were mentioned by adolescents in particular, and were predominantly reported in Kenya. The impact of fear of blood testing for HIV on ANC attendance has been reported elsewhere [28] and the institutionalisation of PMTCT at ANC in Kenya may act as a deterrent for adolescents attending ANC. Another barrier to attending ANC among adolescents was pregnancy disclosure among unmarried adolescents. These factors support similar studies which have identified adolescents as a particularly vulnerable group [24,29], and points to the need for adolescent-friendly services which specifically target and encourage younger women to attend ANC by addressing the issues described above and ensuring confidentiality.

Women in both countries described malaria in pregnancy as dangerous and recognised the risks to themselves and their infants, a finding that is consistent with studies in other countries in East and Southern Africa and The Gambia [30,31]. Pregnant women were seen to suffer from malaria more than others since they were already burdened with carrying a baby, and that malaria in pregnancy harms not one but two people, mother and baby. In Kenya, HIV infection was also seen to predispose people to malaria due to HIV being another blood infection, causing a weakened state. Pregnancy symptoms were said to be similar to malaria symptoms, and significant value was placed on getting a laboratory test to confirm suspected malaria before seeking treatment. In Mali, diagnosis was reported to be as important as treatment of malaria, and if women could not afford both, they would get the diagnostic test and resort to herbal remedies which were cheaper. The anti-plasmodial activity of local plants and herbs is poorly understood within the scientific community [32]. The importance pregnant women in Kenya placed on testing for malaria has not always been the case [30] but was reported in another more recent study [33], and may be a new phenomenon linked to increased awareness of the value of testing and treatment for HIV.

The source of treatment for malaria sought by women was driven by a complex interplay of several factors including severity of the illness, perceived drug safety, drug efficacy and availability, trust in particular providers, convenience and cost, consistent with another study in Kenya [34]. The risks associated with malaria in pregnancy and severe malaria meant women’s preference was to seek diagnosis and drugs from a trained provider at a health centre or hospital. Convenience and cost mitigated their ability to always pursue their preferred choice, using shops in Kenya, or herbal medicines in Mali, both of which were convenient, cheap and sourced locally, and high rates of self-treatment in Mali has been observed in a previous study [35]. Stock-outs of antimalarials at health facilities also resulted in women using alternative sources. These alternative sources were not trusted to provide drugs that were safe and they did not provide a differential diagnosis. Although antimalarials were provided free to pregnant women by the Malian government, having to pay for the partner drugs calls for the government to review and clarify the messages aired on Mali television. Women’s confusion about which drugs were safe to take in pregnancy, and complaints of unpleasant side effects from certain drugs, or that efficacious drugs such as AL were ‘too strong’, which affects compliance [24], suggests that women are not provided with sufficient information about these and other drugs given at ANC. The report from Malian women that eating before taking certain drugs in pregnancy reduced the risk of side effects confirms findings from our health facility survey [16]. There needs to be community education about medications that are safe and unsafe in pregnancy, with a focus on the safe drugs (e.g. SP, AL in second and third trimester, quinine), to remove the notion that some drugs that are effective are too strong to be safe.

Concerning IPTp, women participants in both Kenya and Mali reported having experienced side effects to SP and some voiced concern that SP was no longer effective for treatment of malaria, supporting our findings from household surveys conducted in parallel to this study in Kenya and Mali [17,18]. Although SP is no longer efficacious in these countries for the treatment of malaria, it remains an effective means to prevent malaria through IPTp. Women however did not distinguish between efficacy of SP for prevention and treatment. Our household surveys showed uptake of two doses of IPTp in Kenya and Mali was low, and the proportion of women who received IPTp by DOT was very low, particularly in Mali. This is consistent with the report in Mali that the CSRef told women to eat before taking IPTp-SP, and with our health facility survey where health providers gave this as a reason for not adhering to DOT [16]. Knowledge of malaria and its prevention was a predictor of IPTp receipt and, together with not experiencing any side effects to the previous dose, receipt by DOT in Kenya[18]. Predictors of receiving IPTp by DOT in Mali were being unmarried and living more than 5km from the health facility [17]. Women and men in the FGDs in Mali said that husbands played an important role in determining health seeking practices of pregnant women and paid the costs for women to attend ANC. Previous studies have stated that women need their husbands’ approval before taking drugs in pregnancy [36,37]. Women who live close to a health facility may argue that they need to return home to eat before taking their IPTp dose. Programmes need to ensure that women and household members are provided with accurate information to help them understand the importance of each of the national recommendations: for IPTp, early diagnosis and treatment with safe and efficacious drugs, and ITN use.

ITN use among pregnant women was high in both countries (80% and 93% in Kenya and Mali, respectively) and a high proportion of nets used by pregnant women were said to be obtained from ANC (74% in both countries) [17,18]. As many women in Mali first attend ANC in their 1^st^ trimester (41%), if ITNs are provided at first ANC visit, there is an opportunity for women to receive protection from an ITN use from early pregnancy. The end of the first trimester is a key period during which 65% (95% CrI, 61%–70%) of the potentially infected pregnancies first experience infection, and primigravidae experience a high proportion 39% (95% CrI, 33%–46%) of the total potential malaria-attributable LBW burden [38], hence additional strategies are needed which target women before or early in pregnancy and adolescents. Women in Kenya make their first ANC visit later than in Mali (median of 5 months gestation [IQR 4–6] compared to 4 months gestation[IQR 3–5]) highlighting the need for greater efforts in both countries, and Kenya in particular, to encourage earlier attendance at ANC. The finding that ANC was identified as an important source of nets in both the qualitative and quantitative studies in both countries supports the continued delivery of ITNs to pregnant women through ANC. The report by women in Mali that health providers show favouritism in distributing ITNs may be linked to whether they live in the health facility’s catchment areas, as observed in our health facility survey [16], or may be a legitimate cause for concern.

The FGD findings have several implications for the health service delivery in Kenya and Mali (Table 6).

**Table 6:** Implications of findings for programmes.

**Health system building block**	**FGD Themes**	**Implications for programmes**
*Governance*	Corruption among staff	Introduce systems that promote staff accountability
	Lack of respect to patients	Provide staff with patient-centred training; guidelines and training for assisted deliveries to include provider-client centred approach; performance-based remuneration; provide an anonymous complaints system for clients
*Human resources*	Staff not fully qualified or poorly trained	Deploy only fully qualified staff or ensure that trainees are accompanied or supervised by fully qualified personnel.
	Staff overburdened; long waiting times	Ensure adequate human resources; improve rosters for staff to ensure enough staff are available at busy ANC times
*Service delivery*	Staff applying informal fees for ITNs or antimalarials	Introduce systems that promote staff accountability (see also Governance)
	Administration of drugs in pregnancy	Ensure all providers (pharmacists, private sector clinics and public sector facilities) are trained to ask women of child bearing age their pregnancy status to avoid exposure to ACTs in first trimester
	HIV testing	Ensure that HIV testing involved adequate consenting without undue pressure
*Products and technologies*	Side reactions to drugs	Information for women and households about how to take drugs and which side effects to anticipate
	Information on safety and efficacy of drugs	Information for women and households on the safety and efficacy of drugs
	Seasonal use of ITNs	Information of risks associated with not using an ITN in all seasons, especially in pregnancy
	Stock out of ITNs	Ensure continued supply through improved budgeting, procurement and distribution; avoid diverting nets to population-wide campaigns
*Information systems*	Performance of health staff	Use data on proportion of ANC attendees who get certain services to assess performance for performance-based remuneration
*Financing*	Fees for ANC services	Review of fee structures for ANC (Mali)
	Antimalarial partner drugs are expensive	Governments and partners to review fees for non-antimalarial partner drugs to treat pregnant women and the necessity of prescribing partner drugs

*Governance*: Reports of staff charging unofficial fees for services that should have been provided free, or prioritised clients who were able to pay bribes, calls for systems for ensuring accountability of staff, such as an anonymous complaints system, such as a hotline for clients to report unofficial fees for ACTs as used successfully in Kenya, and advertising government fee policies in national newspapers. To promote respect for ANC clients, the health system would benefit from exploring opportunities for a patient-centred approach to service delivery, and ensuring that the teachers and mentors of medical staff are trained to provide compassionate care during assisted deliveries. Performance-based remuneration which ties their salaries and bonuses to outcome measures should include measures of patient satisfaction and revenue.

*Human resources*: Trainee staff need to be supervised by fully qualified personnel. Ministries of health need to ensure adequate deployment of staff to counteract long waiting times, and district managers should support facility managers to review staff rosters to make sure sufficient qualified staff are on duty during busy ANC clinics.

*Service delivery*: Formal channels for complaints from ANC attendees are needed to report abuse of non-fee paying systems together with performance-based schemes which utilise coverage data for key services, such as proportion of women on their 1^st^ antenatal visit who received an ITN. Peers should be encouraged to report such practices, though that might prove more difficult to enforce.

All health providers in the public, private and commercial sectors as well as pregnant women need information on the correct administration of antimalarial drugs to treat malaria in pregnant women, and prescribers of drugs need to be trained to ask all women of childbearing age whether they suspect they may be pregnant, or to offer a pregnancy test.

Similarly all public and private sector providers should be reminded that HIV testing and counselling should be voluntary.

*Products and technologies*: Information on the safety, efficacy and side effects of drugs used in pregnancy should be provided to pregnant women as part of routine health education at ANC and through media channels that reach all community members. Similarly information on the benefits of ITN use throughout pregnancy, and of IPTp, should be provided.

Stock outs of ITNs were reported in both countries, supporting data from the health facility studies in Mali [15,16]. Although it is not known whether this was a district or nationwide shortage, a continuous supply of ITNs requires adequate budgeting, procurement and distribution of ITNs and a system that ensures ITNs destined for ANC do not get diverted to population-based campaigns. Systems to ensure continuous supplies of antimalarials are also needed, to avoid preventing women from accessing treatment from qualified providers.

*Financing*: The biggest barrier to attending in ANC in both countries was cost; this calls for an urgent review of ANC fee paying structures, and preferably removal of all ANC fees.

### Strengths and limitations

The study draws findings from two countries with very different health system and social contexts to increase the range of perceptions, behaviours and contexts identified, to allow comparison between sites, to explain the quantitative data from our household surveys and to compare these with the findings of our health facility surveys. The research team aimed to encourage openness among participants by holding separate FGDs for women, men, and adolescents, and by using female moderators for the FGDs with women, and male moderators for the FGDs with men. FGD are a technique to understand the normative discourse in a community and the views of people who do not have direct close experience of ANC may differ significantly from real experiences and practices. It is possible that the research teams from KEMRI and MRTC were known to the participants for conducting clinical trials, resulting in a social desirability bias. While FGDs were held in two communities, results should not be considered generalisable to other communities unless they have similar contexts. The broad scope of the guide, which included ANC as well as malaria specific practices, has limited more in-depth discussions on each of the topics such that the emergent themes may not have reached saturation.

## Conclusion

Despite the availability of relatively simple interventions to prevent the adverse consequences of malaria in pregnancy that can be delivered through the ANC platform, access and use is far from optimal. In Kenya and Mali, pregnant women experience substantial barriers to receiving quality care for malaria treatment and prevention, many of which have been found in other settings in Africa. Remedial action needs to be taken jointly by malaria and reproductive health programmes in these countries if coverage of malaria and other ANC interventions is to improve, both the supply and demand for quality services across the antenatal care platform. Access to quality antenatal services is a rights-based issue, and more needs to be done to increase the health service accountability to pregnant women.
